# Changes in mitochondrial function in patients with neuromyelitis optica; correlations with motor and cognitive disabilities

**DOI:** 10.1371/journal.pone.0230691

**Published:** 2020-03-26

**Authors:** Forough Foolad, Fariba Khodagholi, Seyed Massood Nabavi, Mohammad Javan

**Affiliations:** 1 Department of Physiology, Faculty of Medical Sciences, Tarbiat Modares University, Tehran, Iran; 2 Neuroscience Research Center, Shahid Beheshti University of Medical Sciences, Tehran, Iran; 3 Department of Regenerative Biomedicine, Cell Science Research Center, Royan Institute for Stem Cell Biology and Technology, ACECR, Tehran, Iran; 4 Department of Brain and Cognitive Sciences, Cell Science Research Center, Royan Institute for Stem Cell Biology and Technology, ACECR, Tehran, Iran; University College London, UNITED KINGDOM

## Abstract

**Background:**

Neuromyelitis Optica (NMO) is an inflammatory demyelinating disease that mainly affects optic nerves and spinal cord. Besides, loss of motor and cognitive function has been reported as important symptoms of disease.

**Objective:**

Here we investigated the mitochondrial dysfunction and metabolic alterations in NMO patients and evaluate their correlation with disease progress, disability and cognitive impairment.

**Methods:**

The individuals (12 controls and 12 NMO) were assessed for disease severity by expanded disease status scale (EDSS), cognitive function via symbol digit modalities test (SDMT) and fine motor disability by 9-hole peg test (9-HPT). We have measured Sirtuin 1 (SIRT1), SIRT3, mitochondrial complex I, complex IV, aconitase and α-ketoglutarate dehydrogenase (α-KGD) activity in peripheral blood mononuclear cells (PBMCs). Furthermore, SIRT1, pyruvate, lactate and cytochrome c (Cyt c) were determined in plasma.

**Results:**

Our results exhibited increased 9-HPT time in NMO patients. 9-HPT results correlated with EDSS; and SDMT negatively correlated with disease duration and number of attacks in patients. Investigation of PBMCs of NMO patients exhibited a decrease of mitochondrial complex I and IV activity that was significant for complex IV. Besides, complex I activity was negatively correlated with 9-HPT time in NMO group. In the plasma samples, a correlation between pyruvate to lactate ratio and EDSS in NMO patients was found and a negative correlation between Cyt c concentration and SDMT was detected.

**Conclusion:**

Our data support the hypothesis that mitochondrial dysfunction occurred in the CNS and the peripheral blood may contribute to disease progress, disability level and the cognitive impairment in NMO patients.

## 1. Introduction

Neuromyelitis optica (NMO) is known as an autoimmune disorder of the central nervous system (CNS) which attack optic nerve and spinal cord. The myelin of axons located at the optic nerves and spinal cord have been attacked by immune system, thus an extensive inflammation in the optic nerve and spinal cord happens [1]. However, NMO was traditionally considered as a variant of Multiple Sclerosis (MS), but after the discovery of a disease-specific immunoglobulin G antibody (NMO-IgG) that selectively binds to aquaporin-4 (AQP4; the most abundant water channel in the CNS), it is now considered as a distinct disease [2,3]. The most prevalent form of NMO disease shows the relapsing-remitting (RR) course, and the patients experience greater number of attacks than RR-MS patients in a similar follow-up duration. The term NMO spectrum disorders (NMOSDs) was introduced to mention AQP4 IgG-seropositive patients [1].

Although the major lesions and tissue atrophy of NMO patients is in optic nerve and spinal cord, regional brain atrophy has also been reported in this disease [4–6]; for instance, investigations demonstrated that NMO patients suffer cognitive dysfunction [6,7]; with main cognitive deficits in long-term memory, speed of information processing, attention and executive functions.

On the other hand, studies suggested that access of systemic immunoglobulins and lymphocytes into the CNS through a disrupted blood brain barrier (BBB) aggravates disease activity and increases disability and contributes in the CNS lesions in patients [8–10].

Although metabolic profiling can provide an immediate indication of the physiology/pathology of the biological systems; accordingly several studies have characterized the metabolic changes in the CNS and cerebral spinal fluid (CSF) of NMO patients [11,12]. A comprehensive analysis in plasma and peripheral lymphocytes metabolic status and mitochondrial function parameter as well as their relation with disability level and cognitive impairment have not been performed.

Mitochondria are the ubiquitous energy-producing organelles that additionally participate in numerous physiologic and pathologic pathways including apoptosis, reactive oxygen species (ROS) generation and calcium buffering [13]. The electron transport respiratory chain (ETC) proteins are embedded in the inner membrane of mitochondria and tricarboxylic acid (TCA) cycle enzymes activity is located in the organelle matrix provide electrons to drive synthesis of adenosine triphosphate (ATP) molecules by ETC [13]. Mitochondria have significant impact in preserving the structural integrity of myelinated axons, [14], and also, their functional change in peripheral blood mononuclear cells (PBMCs) can influence the fate determination of cells to pro-inflammatory and/or anti-inflammatory [15]. Studies have showed a close relationship between the systemic metabolic status and immune function [16].

In this study, to investigate the relationship between the systemic metabolic and NMO severity; we evaluated the activity of mitochondrial complex I and IV, and also aconitase and α-ketoglutarate dehydrogenase (α-KGD) as important enzymes to energy production in PBMCs of NMO patients. Moreover, we measured the level of sirtuin 1 (SIRT1) and 3 in PBMCs and SIRT1 in plasma due to their critical roles in the physiology of central nervous system, immune system and metabolism [17] and long term changes in gene expression via their epigenetic modifying role. In addition plasma level of pyruvate and lactate was determined to show aerobic/nonaerobic metabolism ratio of CNS, and cytochrome c (Cyt c) level as a marker of apoptosis. Eventually, we assessed the correlation of all results with clinical outcomes to shed light on pathological pathways involved in disability boost and disease progress.

## 2. Methods

### 2.1. Subjects

The study was conducted according to international guidelines and approved by Tarbiat Modares University Ethics Committee. An informed consent was obtained from all patients. Inclusion criteria for NMO group (n = 12) were age between 18 to 66 years, and NMO diagnosis based on the 2015 International Panel for NMO diagnosis (IPND) criteria [18]. All NMO patients were in the remit phase of disease; 8 patients were sero-positive for AQP4 while the rest of them were sero-negative. Information about their treatment content and AQP4 sero-positivity are presented as Supporting Information (S1 Table). Exclusion criteria were clinical relapse, pregnancy, a course of steroids in the last 4 weeks, a history of other autoimmune disorders, vascular disease, or active acute or chronic infections, use of antibiotics in the last 30 days, a history of intracranial or intraspinal tumor, Diabetes or other metabolic disease, or a history of smoking and alcohol or drug abuse. The control group consisted of 12 healthy subjects, matched for age and gender. Exclusion criteria for control group were same as mentioned for NMO patients.

### 2.2. Clinical scales

All subjects were clinically assessed for physical disability using the Extended Disability Status Scale score (EDSS) [19]. For this scale, a higher score indicates more disability. Also upper extremity function and fine motor skills were evaluated with the 9-Hole Peg Test (9-HPT). The test was carried out by having the participant pick up pegs one at a time and place them into the one of the nine holes as quickly as possible. Once all pegs were placed, the pegs must be quickly removed one by one and placed back in the holding place. The test score reflects the time from start of the test to the time the last peg is placed back into the container. Separate trials were held for the dominant and non-dominant hand.

### 2.3. Cognitive function assessment

Cognitive assessment was performed by Symbol Digit Modalities Test (SDMT). This test takes about 5 minutes to administer and is designed to assess speed of information processing [20]. For this test, participants used a key of 9 symbols that each of them was correlated with a single digit. They were asked to write down the digit associated with this symbol as quickly as possible in the page contains symbols with an empty box next to them. Digits and symbols were chosen based on patient’s native speaking language. The outcome score is equal to the number of symbols correctly decoded into the corresponding number within the time permitted.

### 2.4. Collection of plasma and PBMCs

Just after of the clinical assessment, subjects were asked to undergo to a venipuncture for the blood samples. Blood samples were drawn from patients and controls using ethylenediaminetetraacetic acid (EDTA)-containing tubes. Fresh blood samples were processed for isolation of plasma and PBMCs by density gradient centrifuge using Ficoll-Paque (Lymphodex, Inno-Train, Germany).

### 2.5. Measurement of SIRT1 and 3 protein

Total protein was isolated from cells via buffer containing 10 mM HEPES (pH 7.9), 10 mM KCl, and 1.5 mM MgCl2 supplemented with 1 mM PMSF, 2 mM DTT, and 1X proteinase inhibitor. Then protein concentration of samples were determined using Bradford method [21]. SIRT1 level was evaluated from total protein of PBMCs and plasma samples by ELISA kits according to the manufacturer’s guidelines (Abcam, Cambridge, UK). Also, SIRT3 level was determined in extracted protein of PBMCs using commercial ELISA kits (MyBioSource, San Diego, USA).

### 2.6. Mitochondria isolation from PBMCs

PBMCs isolated from patients and the controls were homogenized in buffer containing 20 mM HEPES pH 7.4, 0.2 mM PMSF, 1 mM DTT, 10% glycerol, 2 mM sodium citrate, 1X proteinase inhibitor. For mitochondrial isolation, each samples were homogenized in ice-cold buffer (0.2 mM sodium citrate, 50 mM Tris-HCl, pH 7.4) for 40 s at 210 rpm. Cell homogenate was centrifuged for 10 min (800× g, 4°C). After transferring the supernatant into a new tube, samples centrifuged for 10 min (20,000× g, 4°C). The resulting pellet was re-suspended in ice-cold 0.2 mM sodium citrate and then sonicated for 20 s [22]. Before the enzymatic assays, the extract was further diluted and protein concentration of samples were determined using Bradford method [21].

### 2.7. Complex I activity assay

Mitochondrial complex I activity was assayed according to standard method [23] in KH2PO4 buffer (50mM, pH 7.5), containing 3.75 mg/ml fatty acid-free BSA and 0.1mM decylubiquinone. To initiate the reaction 0.1 mM NADH was added. To determine the background rate, parallel measurements in presence of Rotenone (5μM) were used. The absorbance was monitored at 340 nm (ε = 6.22mM-1.cm-1).

### 2.8. Complex IV activity assay

For assessment of complex IV activity, the oxidation of Cyt c was monitored at 550 nm (ε550 = 18.5 mM-1.cm-1) according to the method described by Spinazzi et al. [24]. The reaction buffer was composed of 50 mM potassium phosphate, pH 7.0, and homogenate proteins. The reaction was initiated at 37°C by the addition of 60 μM reduced Cyt c.

### 2.9. Aconitase activity assay

Aconitase catalyzes an equilibrium between aconitate, cis-aconitate and iso-citrate, and cis-aconitate production is proportional to aconitase activity. The activity of aconitase in PBMCs was measured using a Tris-HCL buffer (pH 8.0, 100 mM) supplemented with 20 mM D,L-trisodium isocitrate, and 0.6 mM fresh MnCl_2_. The reaction was initiated by the addition of mitochondria. Changes in the absorbance were monitored at 240 nm using a microplate reader (Biotek, Synergy HTX) and calculated by an extinction coefficient of 3.6 mM-1.cm-1 [25].

### 2.10. Alpha-ketoglutarate dehydrogenase activity assay

Alpha-ketoglutarate dehydrogenase (α-KGD) is a Krebs cycle enzyme, which catalyses the non-equilibrium reaction converting α-ketoglutarate, coenzyme A and NAD+ to succinyl-CoA, NADH and CO2, requiring thiamine pyrophosphate as a cofactor. α-KGD activity was measured based on method by Gibson and colleagues [26]. The reaction medium included assay buffer containing 50 mM Tris (pH 7.0), 1 mM MgCl2, 1 mM CaCl2, 0.5 mM K-EDTA, 1 mM dithiothreitol, 1% triton X-100, and also 0.3 mM thiamine pyrophosphate, 1 mM NAD, 0.163 mM coenzyme A and the sample (25–30 μg protein). Then 1.25 mM 2-ketoglutarate was added to the reaction medium, when the baseline stabilized at 37°C. The formation of NADH was monitored at 340 nm (ε340 = 6.23 mM-1.cm-1).

### 2.11. Determination of pyruvate level

Plasma samples were mixed to assay buffer containing 0.25 M Tris buffer (pH 7.5) and 0.25 mM NADH. Baseline absorbance of samples was measured at 340 nm. After that, 3μl LDH (3 mg/ml) was added to each sample and incubate for 2 min. finally, absorbance was re-measured at 340 nm. Calculation was done by a standard curve [27].

### 2.12. Measurement of lactate level

The determination of lactate was carried out following the method described by Marbach and Weil [27]. Plasma samples were added to assay mixture containing 0.43 M glycine-hydrazine buffer, 2.5 mM NAD and 3μl lactate dehydrogenase (LDH) (3 mg/ml). Then the mixture was left to stand at ambient temperature for 14 min and the absorbance was read at 340 nm. The level of lactate was calculated by a standard curve.

### 2.13. Detection of Cyt c level

Level of cytochrome c was estimated based on a method introduced by Williams [28]. Each sample was loaded in 2 separate wells. One well was oxidized by hydrogen peroxide and the other was reduced by sodium dithionate. Finally the spectrum of well 2 vs. 1 was recorded from 535 to 550 nm and the results were calculated by the extinction coefficient of 19 mM-1cm-1.

### 2.14. Statistical analysis

The Kolmogorov–Smirnov Test was used to assess the normal distribution of data. For normally distributed data, groups difference significance was assessed by Student's t-test for unpaired observations and presented as mean±SEM. Nonparametric data were analyzed and presented as median±IQR. In order to explore correlation between variables, Pearson’s correlation (r) for parametric data and Spearman’s correlation (r) for nonparametric results were used. The statistical significance was established when p-value was less than 0.05.

## 3. Results

### 3.1. Clinical variables of subjects

The demographic and clinical features of control healthy subjects and NMO patients are summarized in Table 1. In NMO patients, the female to male ratio was 8:4, the mean age of patients was 40.1 ± 2.7, the mean duration of disease was 7 ± 1.2, the number of attacks was 2.2 ± 0.3, and mean EDSS score was 1.6 ± 0.6. The Mean age of control subjects was 38.7 ± 2.4.

**Table 1:** Clinical neurological data of neuromyelitis optica (NMO) patients and age-matched controls.

	Controls (n = 12)	NMO (n = 12)
**Gender**	F 9: M 3	F 8: M 4
**Age (years)**	38.7 ± 2.4 (29–54)	40.1 ± 2.7 (26–58)
**Disease duration (years)**	N/A	7 ± 1.2 (1–15)
**Number of attacks**	N/A	2.2 ± 0.3 (1–5)
**EDSS**	N/A	1.6 ± 0.6 (0–6)

Values are expressed as mean ± SEM. NMO = Neuromyelitis optica; F = female; M = male; EDSS = Expanded disability status scale; N/A = Not Applicable.

Seven patients were lesion free in their MRI, 5 patients had lesions characteristically occurred in the deep white matter, periventricular and Juxtacortical regions that were non-specific for MS. The presence and/or absence of brain lesions for each patient are shown in Supporting Information (S1 Table).

### 3.2. Fine motor function but not cognitive function was affected by NMO disease

**Fig 1:**
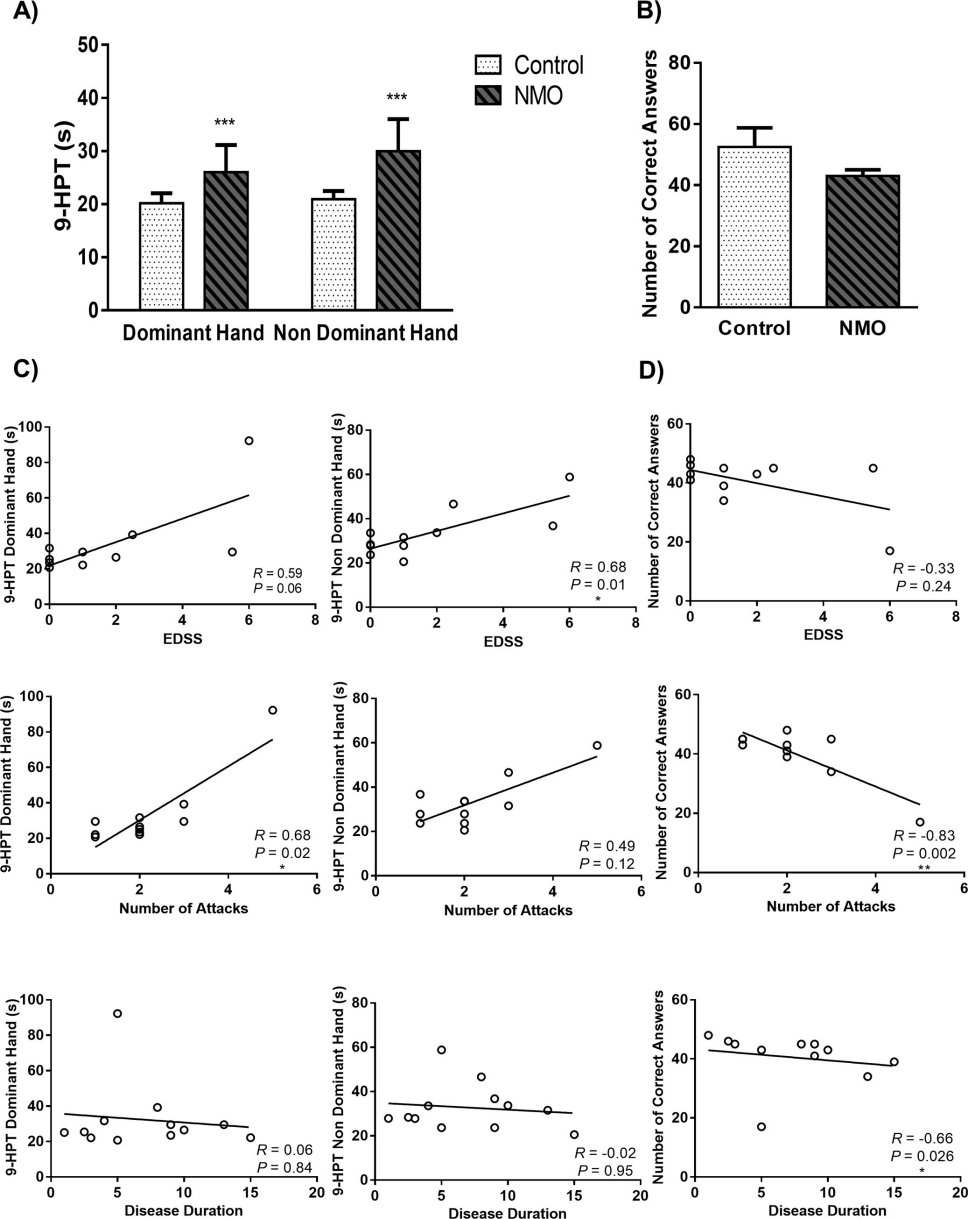
Dominant hand and non dominant hand time of 9-Hole Peg Test (9-HPT) **(a)** and Symbol Digits Modalities Test (SDMT) scores **(b)** of Neuromyelitis Optica (NMO) patients compared to control group. Each point shows the median ± IQR. (***P<0.001 different from the control group). The correlation of 9-HPT **(c)** and SDMT **(d)** with the expanded disease status scale (EDSS), disease duration and number of attacks in NMO patients.

9-HPT was done to measure disability level in fine motor function. A comparison between control group and patients showed a significant increase in dominant hand and non-dominant hand time to finish the task in NMO group (Fig 1A). The time performance of NMO patients revealed an increase about 1.6 and 1.5 fold, in dominant hand and non-dominant hand, respectively. 9-HPT results were significantly correlated with some clinical scales (Fig 1C). The correlation was stronger with EDSS (DH *R* = 0.59, *P* = 0.06; NDH *R* = 0.68, *P* = 0.01) followed by number of attacks (DH *R* = 0.68, *P* = 0.02; NDH *R* = 0.49, *P* = 0.12); but no significant correlation with the disease duration (DH *R* = 0.06, *P* = 0.84; NDH *R* = -0.02, *P* = 0.95) was observed.

SDMT assesses key neurocognitive functions that underlie many tasks, including attention, visual scanning, and motor speed [29]. Our results in Fig 1B showed that cognitive function does not significantly impaired in NMO group. The number of correct answer in an interval of 90 s decreased by 83.8% in NMO patients. Based on cognitive assessment of NMO patients (Fig 1D), number of correct answers were negatively correlated with the number of attacks (*R* = -0.83, *P* = 0.002) and disease duration (*R* = -0.66, *P* = 0.02), but the result did not show any significant correlation with EDSS (*R* = -0.33, *P* = 0.24).

### 3.3. The level of SIRT1 and SIRT3 in were not affected by NMO disease

**Fig 2:**
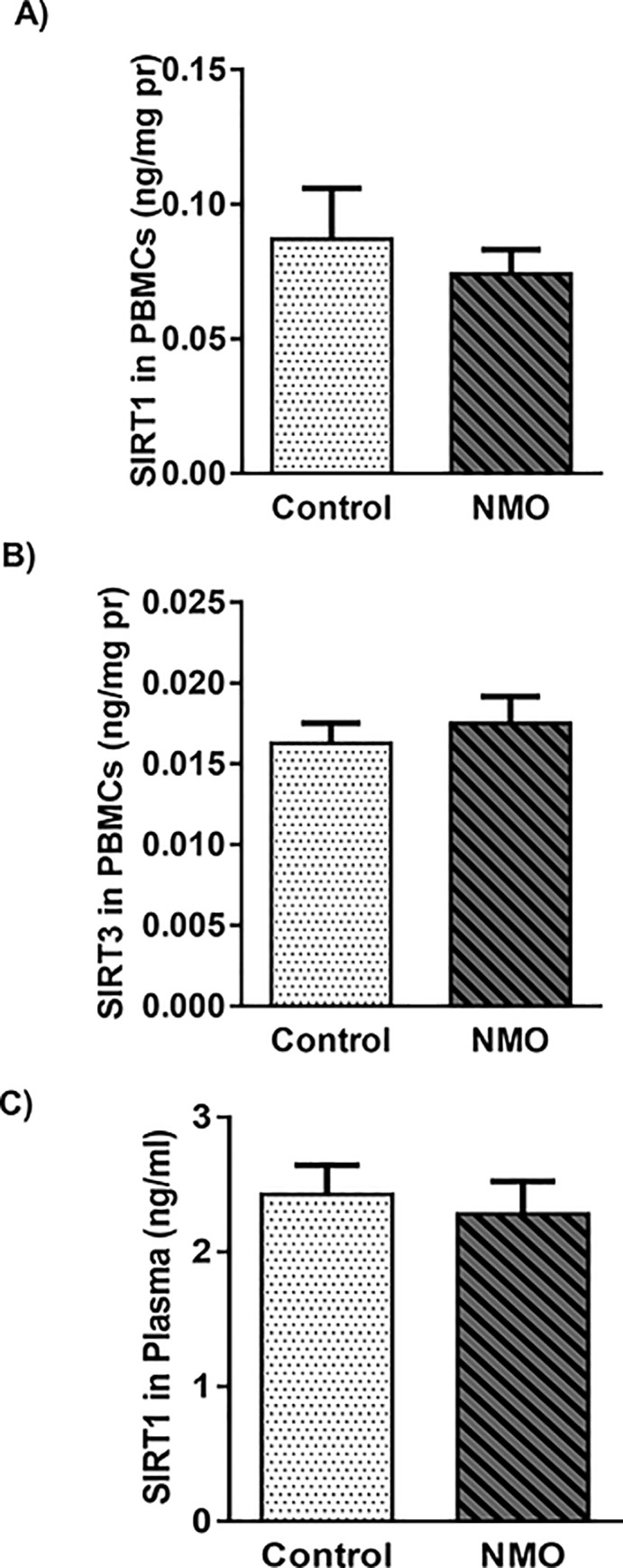
Sirtuin 1 (SIRT1) **(a)** and SIRT3 **(b)** level in peripheral blood mononuclear cells (PBMCs) and SIRT1 level in plasma **(c)** of Neuromyelitis Optica patients compared to control group. Each point shows the median ± IQR **(a)** and/or mean ± SEM **(b** and **c)**.

SIRT1 is a histone deacetylase that regulates the acetylation of a number of cellular substrates, resulting in modification of pathways involved in gene expression and DNA damage repair [30]. As we shown in Fig 2A and 2C the level of SIRT1 in protein extract of PBMCs and plasma, respectively, did not show any changes in NMO group compared to control.

Furthermore, SIRT3 as a mitochondrial deacetylase controls many aspects of mitochondrial function by deacetylating a number of mitochondrial matrix proteins, including anti-oxidant effectors and proteins involved in the ETC, thus limiting the production of ROS [31]. We monitored the level of this protein in PBMCs and the comparison of control and patient samples didn’t show any changes (Fig 2B).

### 3.4. The mitochondrial complexes activity decreased in NMO patients

**Fig 3:**
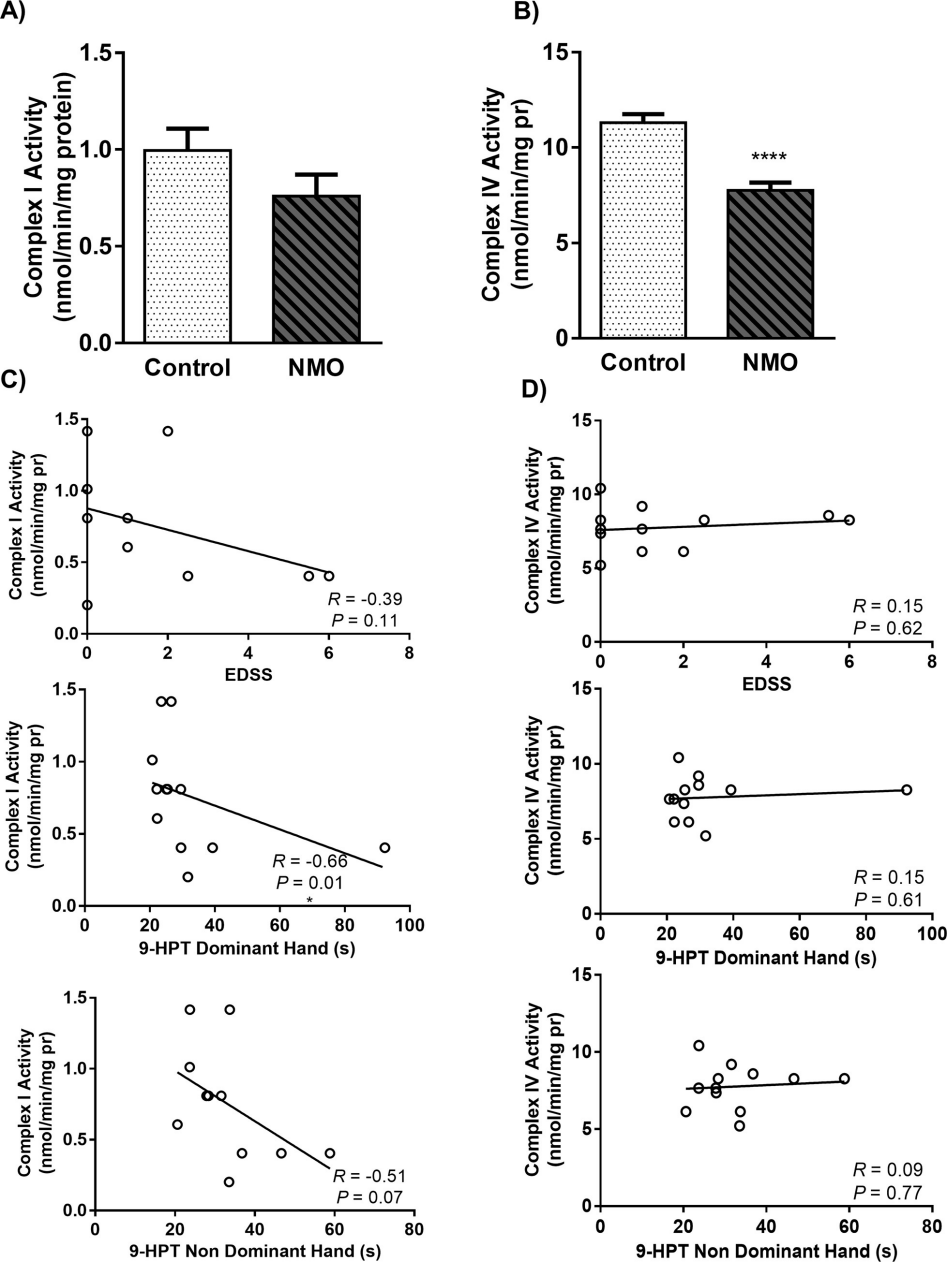
Mitochondrial complex I **(a)** and complex IV **(b)** activity in peripheral blood mononuclear cells (PBMCs) of Neuromyelitis Optica (NMO) patients compared to control group. Each point shows the mean ± SEM. (****P<0.0001 different from the control group). The correlation of Complex I **(c)** and Complex IV **(d)** activity in PBMCs with the expanded disability status scale (EDSS) and 9-Hole Peg Test (9-HPT) in dominant hand and non dominant hand of NMO patients.

Complex I is a large size enzyme that catalyze the first step of the mitochondrial ETC. The enzyme oxidizes NADH transferring electrons to Ubiquinone, a lipid soluble electron carrier embedded in the lipid bilayer of the inner mitochondrial membrane [32]. Besides, Mitochondrial complex IV or Cyt c Oxidase (COX) is the last electron acceptor of the respiratory chain which reduces oxygen to water [33]. Fig 3A and 3B show the mitochondrial complex I and IV activity for mitochondria isolated from PBMCs of subjects. The activity of both complex I and IV were decreased about 76.3% and 68.6%, respectively, in NMO patients. However, just the reduction of mitochondrial complex IV activity was statistically significant.

As shown in Fig 3C and 3D, in order to investigate the correlation of mitochondrial complex I and IV activity with disease progression, we used EDSS and 9-HPT results. The results showed a weak correlation of complex I activity with some of these clinical scales. The correlation was significant with 9-HPT (DH *R* = -0.66, *P* = 0.01; NDH *R* = -0.51, *P* = 0.07) and insignificant with EDSS (*R* = -0.39, *P* = 0.11). The activity of complex IV didn’t show any correlation with EDSS (*R* = 0.15, *P* = 0.62) and 9-HPT (DH *R* = 0.15, *P* = 0.61; NDH *R* = 0.09, *P* = 0.77).

### 3.5. TCA cycle enzymes activity and Cyt c level didn’t change in NMO patients, although the level of Cyt c correlated with cognitive function

**Fig 4:**
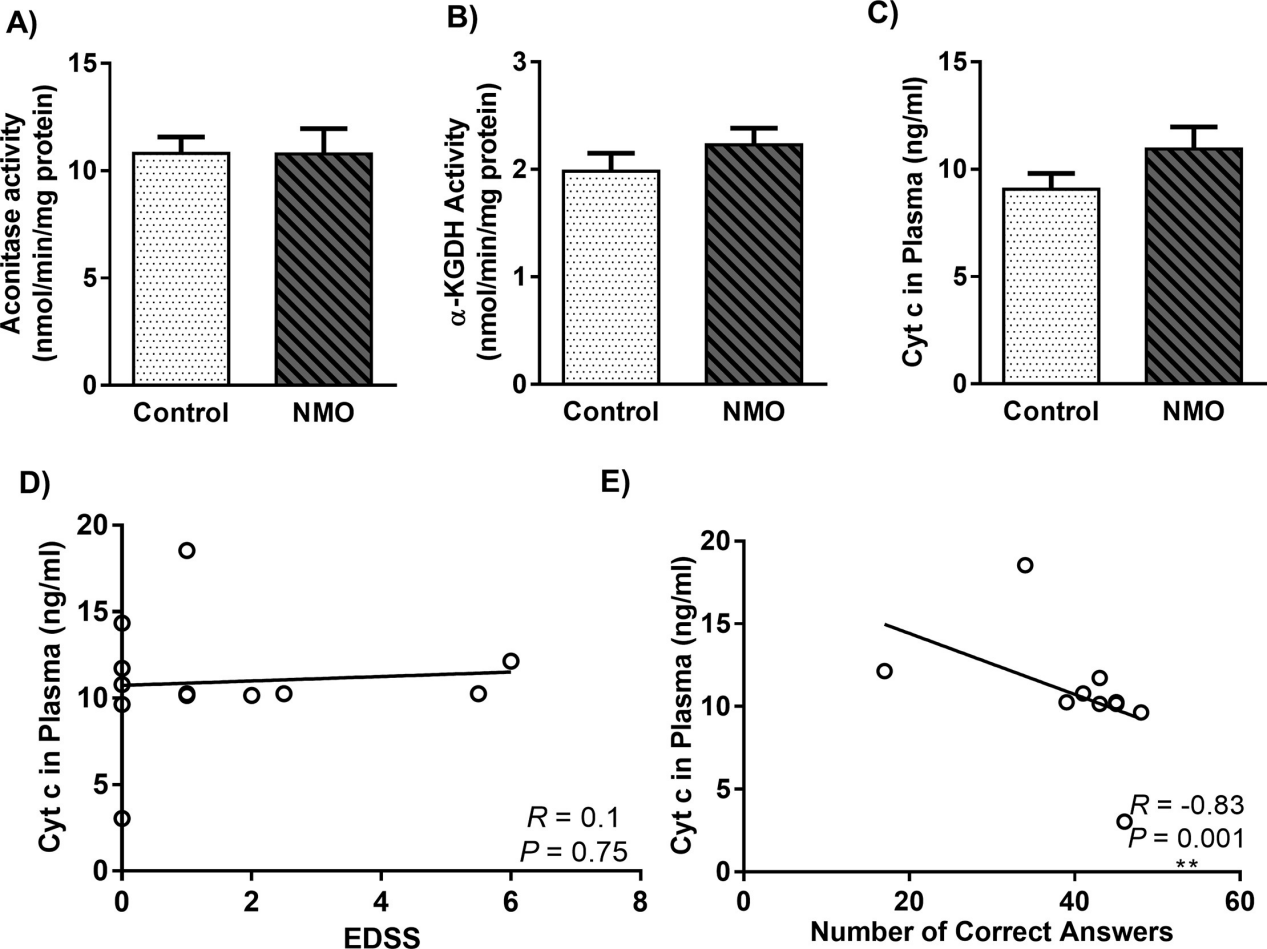
Aconitase **(a)** and α-ketoglutarate dehydrogenase **(b)** activity in peripheral blood mononuclear cells (PBMCs), and Plasma level of Cytochrome c (Cyt c) **(c)** of Neuromyelitis Optica (NMO) patients compared to control group. Each point shows the mean ± SEM. The correlation of Cyt c level in plasma with the EDSS **(d)** and Symbol Digit Modalities Test (SDMT) **(e)** in NMO patients.

Because of the majority role of TCA cycle enzymes in ATP production and cell metabolism, any changes in its elements’ activity could affect cell function, widely. Aconitase enzyme is the most sensitive enzyme to ROS [34]. Also, Alpha-KGDH is a highly regulated enzyme, which could determine the metabolic flux through the Krebs cycle [35]. Based on Fig 4A and 4B, there were no significant differences in aconitase and α-KGD activity in NMO patients compared to healthy controls.

On the other hand, since Cyt c releases from dying cells, we measured the level of this marker as an indicator of neuronal death in NMO (Fig 4C). The level of Cyt c elevated in the plasma of NMO when compared with control group, but non-significantly. As we shown in Fig 4D, the level of Cyt c didn’t correlate with EDSS (*R* = 0.1, *P* = 0.75), while negatively correlated with number of correct answers of SDMT (*R* = -0.83, *P* = 0.001) during NMO disease (Fig 4E).

### 3.6. The ratio of pyruvate to lactate correlated with EDSS

**Fig 5:**
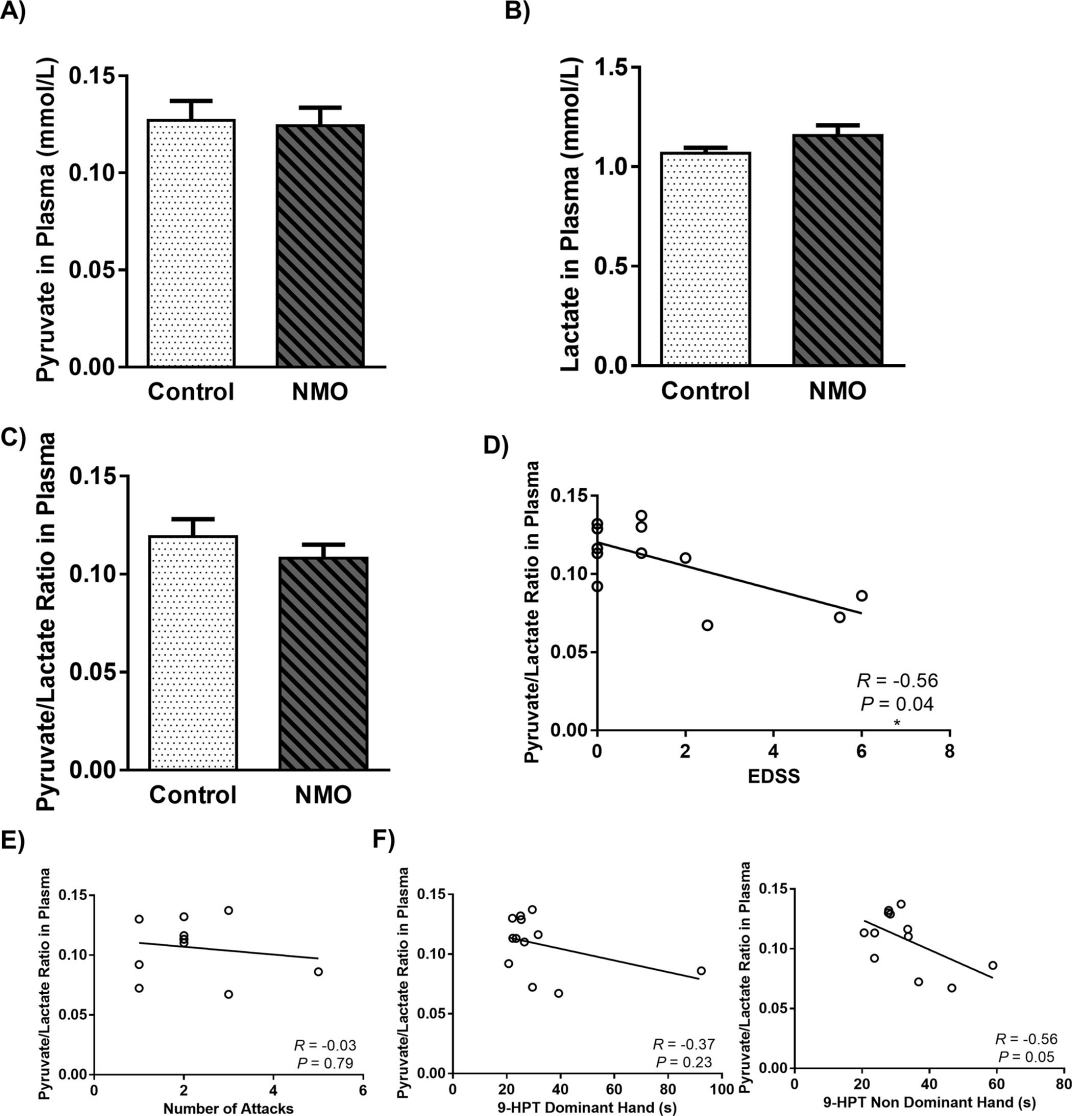
Level of pyruvate **(a)**, lactate **(b)** and pyruvate to lactate ratio **(c)** in Plasma of Neuromyelitis Optica (NMO) patients compared to control group. Each point shows the mean ± SEM. The correlation of Pyruvate to lactate ratio in plasma with the expanded disease status scale (EDSS) **(d)**, number of attacks **(e)** and 9-Hole Peg Test (9-HPT) in dominant hand and non dominant hand **(f)** of NMO patients.

The blood pyruvate to lactate ratio reflects the equilibrium between substrate and product of the reaction catalyzed by lactate dehydrogenase. This ratio is correlated with the cytoplasmic NAD+: NADH ratio and is used as a surrogate measure of the cytosolic oxido-reduction state [36]. We monitored the level of lactate and pyruvate in plasma of all individuals and based on Fig 5A, 5B and 5C, the level of this agents did not show any significant changes in NMO group. However, an insignificant increased in plasma level of lactate and decreased in pyruvate to lactate ratio were indicated in NMO patients. As we shown in Fig 5D, plasma ratio of pyruvate to lactate was correlated with EDSS (*R* = -0.56, *P* = 0.04) in NMO patients. However, there wasn’t any significant relationship between this ratio and number of attacks (*R* = -0.03, *P* = 0.79) (Fig 5E) and 9-HPT (DH *R* = -0.37, *P* = 0.23; NDH *R* = -0.56, *P* = 0.05) (Fig 5F).

## 4. Discussion

In this study we measured different read outs in NMO patients including disease severity (EDSS), fine motor function (9-HPT) and cognitive performance (SDMT). A significant decrease in fine motor performance and a trend for declined SDMT was observed. These finding may be explained by lesion distribution in spinal cord of NMO patients but not extensive lesion in cerebral cortex [37,38].

EDSS was correlated with the number of attacks but not with the disease duration which may imply for the importance of attack prevention in NMO to prevent the disease progression. In a same observation, both 9-HPT and SDMT were also correlated with number of attacks, but not with disease duration. Again it suggests that preventing disease attacks may contribute to better motor and cognitive outcomes in NMO. This will be more notable in the light of the fact that the NMO patients experienced greater attacks in number and severity even compared to MS patients [39]. Several studies have revealed cognitive impairment in NMO patients [6,39]. Here, the decline in SDMT as an indicator of cognitive function was not statistically significant, however, the significant relationship between number of correct answer of SDMT and the number of attacks may indicate a performance reduction in some tasks including attention, visual scanning, and motor speed during the disease progression. These finding may also imply for the significance of cognitive impairment and restorative strategies in NMO patients with higher number of attacks.

Unlike number of attacks, EDSS did not have a significant correlation with SDMT that may suggest cognitive dysfunction may proceed independent of EDSS by recurrent attacks of disease. In the other word cognitive dysfunction may progress while EDSS seeds to be well controlled. Our results corroborate the findings observed in previous studies that demonstrated a relationship between number of relapses and brain tissue volume in NMO disease [40,41]. Both motor dysfunction and cognitive impairment have major impact on life quality of NMO patients; therefore regular evaluation of cognitive performance in NMO patients as like as the EDSS seems required.

Additionally in the present study, metabolic alteration in peripheral lymphocytes, plasma and mitochondria of PBMCs was compared between NMO patients and healthy controls. NMO like other CNS autoimmune diseases, shows different aspects of immune cell activation, BBB disruption and immune cell infiltration into the CNS, evaluation of this cells may be an important approach to study disease pathology and find potential therapeutic targets. With this purpose, we studied metabolic changes in PBMCs and also plasma samples as body fluid that exposed to both neurons and immune cells. We also checked how this measurable biochemical factors may correlate with motor and cognitive dysfunctions. Exploring biomarkers that might correlate with patient’s disability and cognitive impairment in NMO, will help to predict motor and cognitive function separately. Therefor we assessed the correlation of each marker with clinical results.

The crucial role of SIRTs as a family of histone deacetylase that can modulate the activity of numerous proteins and transcriptional activities for long term, has been demonstrated in immune system, metabolism and CNS physiology. SIRTs are known as a molecular link between immunity and metabolic pathways [42]. Although pervious experiments in MS patients, as most close disease to NMO, displayed an elevated level of SIRT1 in the plasma and a decreased level of this protein in PBMCs of MS patients [43,44], our investigation didn’t show any changes in the level of this protein during NMO disease. We measured the level of SIRT1 as suppressor of transcriptional activity of NF-κB [45,46] in plasma and PBMCs, and also SIRT3 as a major mitochondrial deacetylase and metabolic sensor responding to the alteration of energy status in the PBMCs. However, we did not observe a correlation of SIRT1 and SIRT3 level with none of EDSS, number of attacks, 9-HPT and SDMT. Therefore, other mechanisms might be involved in the failure of both motor and cognitive function.

For more specific evaluation of metabolic status in PBMCs, we assessed the activity of mitochondrial complex I and IV, and also aconitase and α-KGD in these cells. Our data suggested a reduction in mitochondrial complexes activity but not in TCA enzymes. The failure of activity in mitochondrial complexes leads to decreased ATP production that might be correlated with pathogenesis activity of lymphocytes. Additionally, the results showed a slight correlation between the reduction of mitochondrial complex I activity and increased time of 9-HPT. One recent study indicated a decreased protein level of mitochondrial complexes in PBMCs isolated from MS patients [47]. It seems that decrease in available energy in cells can affect the pro-inflammatory activity of lymphocytes. Clinical and laboratory-based evidence has shown that in contrast to MS disease, B cells play a prominent role in NMO pathogenesis. B cell or plasma cell-produced NMO-IgG binding to astrocytic AQP4 activates complement deposition [48–50], leading to astrocyte damage and inflammatory reaction [51] with leukocyte infiltration [50] and cytokine release [52], thus resulting in disease development. Therefore, the observed mitochondrial failure worth to be evaluated in different cell types. Some treatments of NMO patients such as steroids and immunosuppressants may affect mitochondrial function; all NMO patients in this study were prescribed azathioprine and mycophenolate mofetil at least for 3 months prior to sampling (see Supporting Information, S1 Table). These drugs are mainly known to inhibit lymphocyte proliferation via purine synthesis prevention [53,54].

In order to test the hypothesis that mitochondrial dysfunction and metabolic changes might be involved in neuronal death and increased disability in NMO patients, we chose some body fluid biomarkers including pyruvate, lactate and Cyt c. Monitoring the mentioned markers may reflect the metabolic changes from an aerobic to a nonaerobic status and also the rate of cell death. Although our investigation didn’t show any significant changes neither in pyruvate nor in lactate level, we found an insignificant increase in lactate level and decrease in pyruvate to lactate ratio of plasma in NMO patients. In addition, the reduction of pyruvate to lactate ratio was negatively correlated with increase of EDSS in NMO group. This related changes and shift from aerobic to anaerobic metabolism may be a reflection of balance destruction in metabolic status of mitochondria in the CNS and its association with neuronal loss. Previous investigations showed a direct relationship between neuronal lose and dysfunction of aerobic energy metabolism [55]. Although, the exact source of these changes is not clear, a previous study has showed an elevated level of lactate in CSF of NMO patients as well [12]. Furthermore, measurement of plasma level of Cyt c displayed an slight but non-significant increase in NMO group, while the level of this protein was in normal range (<25 ng/ml) [56] in both normal and NMO individuals. Interestingly, results analysis displayed a significant negative correlation between elevated level of Cyt c and decreased number of correct answers in SDMT by NMO patients. It has been previously described that Cyt c is not only released from mitochondria, but also, it leaves the cell and can be considered as a biochemical indicator of apoptosis [57,58]. So this slight elevation in the plasma level of Cyt c might be attributed to demyelinated neurons that triggered for apoptosis and led to both brain and spinal cord atrophy reported in NMO disease [6,37,39]. On the other hand, astrocytic damage in the CNS of NMO patients was reported; even it was reported that it to be more severe than demyelination [59]. So it’s worth noting that some parts of these metabolic changes, mitochondrial dysfunctions and elevated apoptosis be related to astrocytes damage in NMO patients.

## 5. Conclusion

Taken together, our results may help to shed light on important change in metabolic status, cognitive impairment and mitochondrial dysfunction in NMO pathogenesis, in both side of the NMO pathology, neuronal loss and inflammatory activity of lymphocytes. In addition, our data indicated the correlation of some mentioned markers with disability and cognitive status that have profound impact on life quality of NMO patients. Also presented data may help to find biomarkers for clinical monitoring and potential therapeutic targets. Although, it seems that metabolic changes can influence disease course, further studies especially in acute stage of NMO are needed to introduce reliable biomarkers for disease course monitoring including the cognitive aspects.

## Supporting information

S1 Table Clinical neurological data of all neuromyelitis optica (NMO) patients. (PDF)

S1 Fig The correlation Symbol Digits Modalities Test with the presence or absence of brain lesions in NMO patients. 0 = Absence of brain lesions, 1 = Presence of brain lesions.
(PDF)

S2 Fig Device prepared for nine hole peg test (9-HPT) as set for checking the patient performance with right hand. (PDF)

S3 Fig A picture from form prepared for Symbol Digit Modalities Test (SDMT). The numbers (1 to 9) in patients native language, Persian, were matched with different symbols.
(PDF)
