# Supplementary material for: Changes in mitochondrial function in patients with neuromyelitis optica; correlations with motor and cognitive disabilities
Source: PLoS One. 2020 Mar 26;15(3):e0230691. doi: 10.1371/journal.pone.0230691 (PMC7098571; doi:10.1371/journal.pone.0230691)
Supplement: S1 Table — (PDF) [file pone.0230691.s001.pdf]

S1 Table Clinical neurological data of all neuromyelitis optica (NMO) patients.

NO	Sex	EDSS	Age (year)	DD (year)	NA	AQP4	Brain lesions	Treatment
**1**	Male	0	35	2.5	2	Positive	Negative	AZA
**2**	Male	2	51	10	2	Positive	Positive	AZA
**3**	Male	0	50	9	2	Negative	Negative	AZA
**4**	Female	5.5	36	9	1	Negative	Negative	AZA
**5**	Female	0	31	5	1	Positive	Negative	AZA
**6**	Female	1	36	13	3	Positive	Positive	AZA
**7**	Female	1	40	15	2	Positive	Negative	MMF
**8**	Female	0	26	1	2	Positive	Negative	AZA
**9**	Female	1	43	3	1	Negative	Negative	AZA
**10**	Female	0	58	4	2	Positive	Positive	MMF
**11**	Male	2.5	43	8	3	Positive	Positive	AZA
**12**	Female	6	32	5	5	Negative	Positive	AZA

NO= number of; EDSS= Expanded disability status scale; DD= disease duration; NA= number of attack; AQP4= Aquaporin-4; AZA= Azathioprine; MMF= Mycophenolate mofetil.
