# Supplementary material for: Changes in mitochondrial function in patients with neuromyelitis optica; correlations with motor and cognitive disabilities
Source: PLoS One. 2020 Mar 26;15(3):e0230691. doi: 10.1371/journal.pone.0230691 (PMC7098571; doi:10.1371/journal.pone.0230691)
Supplement: S1 Fig — 0 = Absence of brain lesions, 1 = Presence of brain lesions. (PDF) [file pone.0230691.s002.pdf]

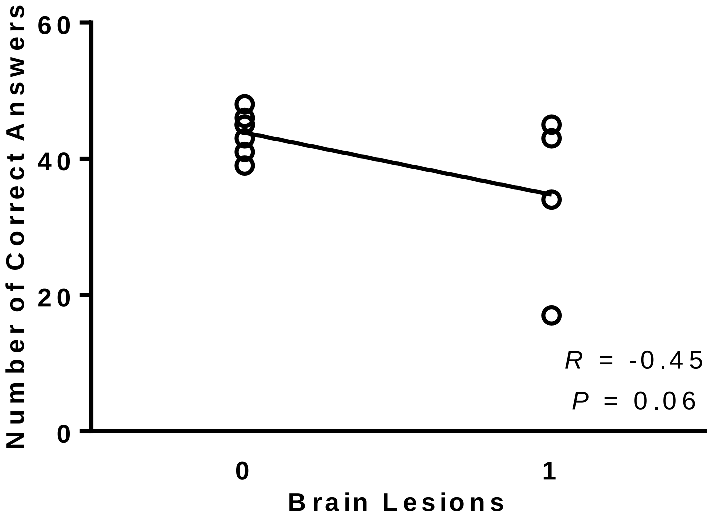

S1 Fig The correlation Symbol Digits Modalities Test with the presence or absence of brain lesions in NMO patients. 0= Absence of brain lesions, 1= Presence of brain lesions
