# Supplementary material for: Changes in mitochondrial function in patients with neuromyelitis optica; correlations with motor and cognitive disabilities
Source: PLoS One. 2020 Mar 26;15(3):e0230691. doi: 10.1371/journal.pone.0230691 (PMC7098571; doi:10.1371/journal.pone.0230691)
Supplement: S2 Fig — (PDF) [file pone.0230691.s003.pdf]

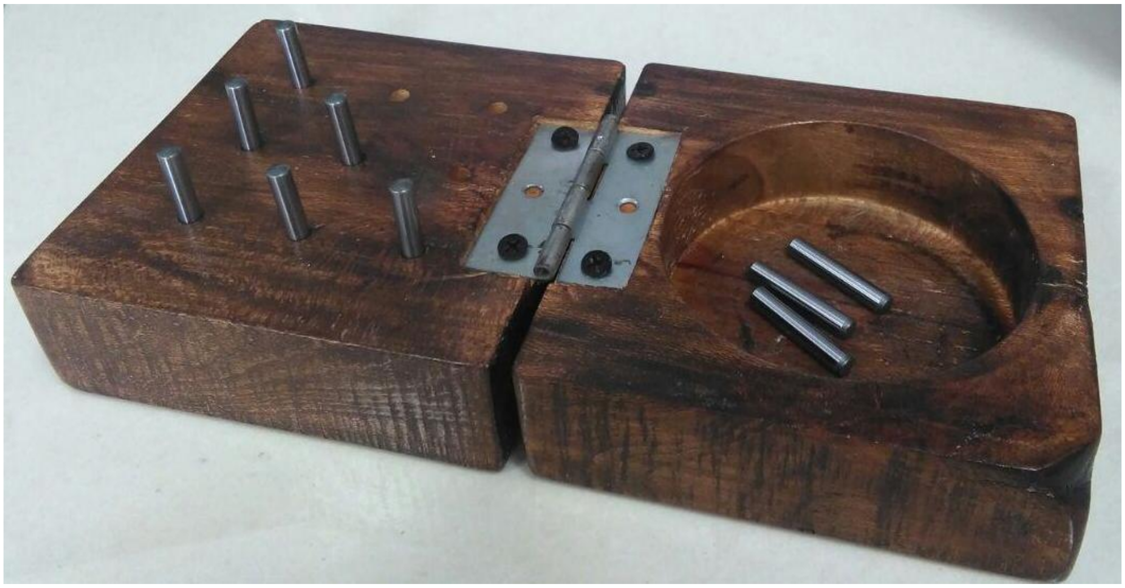

S2 Fig Device prepared for nine hole peg test (9-HPT) as set for checking the patient performance with right hand.
