# Supplementary material for: Changes in mitochondrial function in patients with neuromyelitis optica; correlations with motor and cognitive disabilities
Source: PLoS One. 2020 Mar 26;15(3):e0230691. doi: 10.1371/journal.pone.0230691 (PMC7098571; doi:10.1371/journal.pone.0230691)
Supplement: S3 Fig — The numbers (1 to 9) in patients native language, Persian, were matched with different symbols. (PDF) [file pone.0230691.s004.pdf]

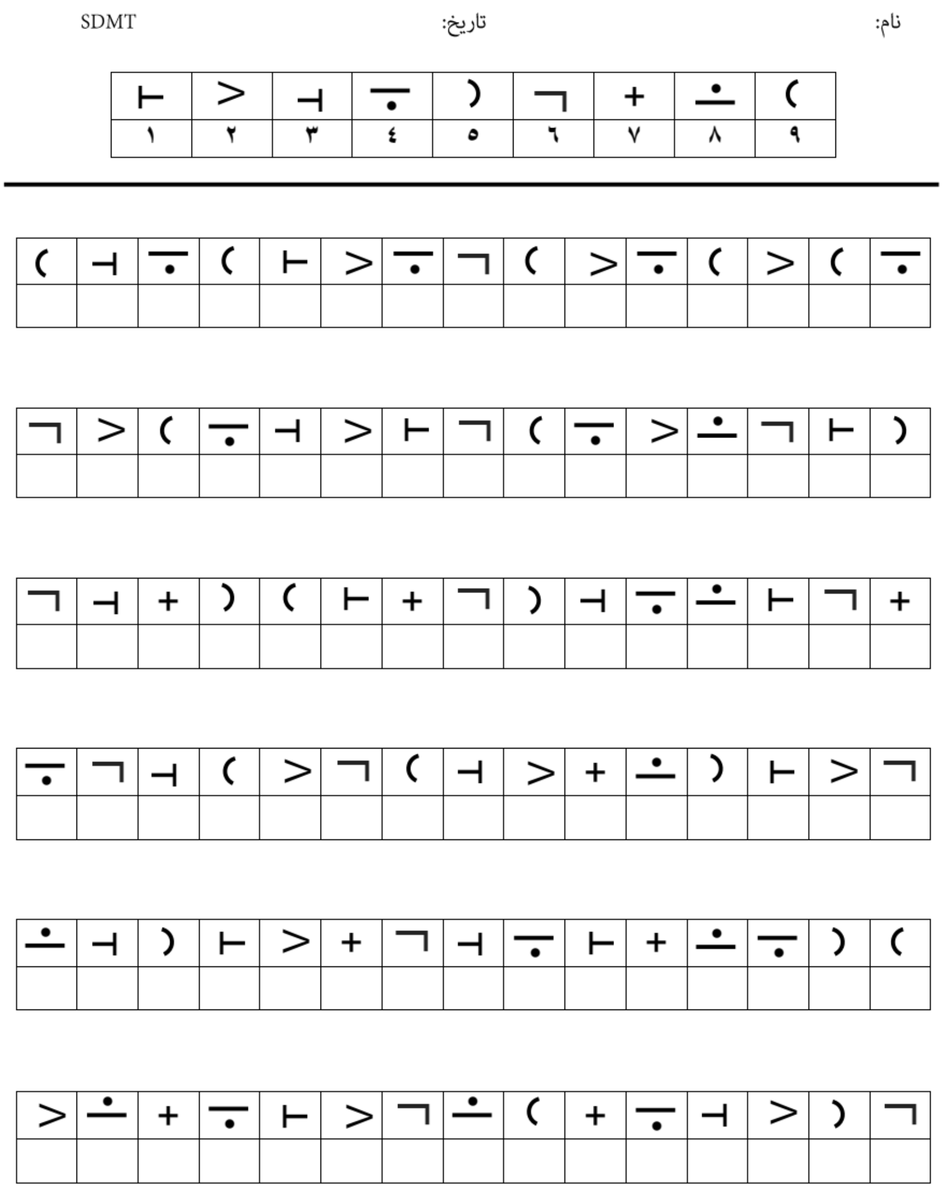

S3 Fig A picture from form prepared for Symbol Digit Modalities Test (SDMT). The numbers (1 to 9) in patients native language, Persian, were matched with different symbols.
